# Facilitating research participation through a research navigator programme: researchers’ insights from a project with asylum seekers and refugees

**DOI:** 10.1186/s13031-025-00729-2

**Published:** 2025-12-02

**Authors:** A. Hägg Martinell, M. Tyrrell, H. Eriksson, M. Mazaheri

**Affiliations:** 1 Department of Health Promoting Science, Sophiahemmet University, Stockholm, Sweden; 2 Department of Nursing Science, Sophiahemmet University , Stockholm, Sweden; 3 Department of Neurobiology Care Sciences and Society, Karolinska Institutet, Stockholm, Sweden; 4 Department of Health Sciences, University West, Trollhättan, Sweden

**Keywords:** Asylum seekers, Community-based research navigator programme, Experience, Qualitative research, Refugees, Research navigator, Thematic analysis

## Abstract

**Background:**

Underrepresentation of vulnerable populations, such as asylum seekers and refugees, remains a persistent challenge in health research, often due to barriers such as limited access, mistrust, and logistical constraints. The study explored researchers’ experiences of using community-based research navigators to facilitate participation among asylum seekers and refugees in a research project conducted in Sweden.

**Methods:**

A descriptive qualitative design was employed, involving eight in-depth interviews with four researchers before and after implementing the community-based research navigator programme. The community-based research navigator programme recruited volunteers from the target population, including Syrian, Eritrean, and Somalian refugees and asylum seekers to support participation in the research project, in Sweden. Data were analysed using reflexive thematic analysis guided by Braun and Clarke’s six-phase framework.

**Results:**

The researchers experienced the community-based research navigators as a ‘critical enabler of participant recruitment and recognition’, while expressed ‘complexities of intergroup relationships and logistics’ which are the two main themes of the results. Navigators’ meaningful engagement and recognition and community-based language concordance as a trust builder are the two subthemes of critical enabler of participant recruitment. Complexities of intergroup relationships and logistics is manifested in three subthemes of power dynamics, communication and relational challenges and logistic challenges.

**Conclusion:**

The community-based research navigator programme showed to be a valuable yet complex strategy for facilitating research participation among asylum seekers and refugees, according to the participating researchers. It also may involve ethical and logistical challenges that require careful planning and sensitive implementation. Tailored training, culture-sensitive approaches, and recognition of intergroup dynamics are essential for enhancing the similar programme’s effectiveness. This approach holds promise for improving inclusivity and equity in health research involving hard-to-reach populations, such as asylum-seekers and refugees.

## Background

A significant limitation across a large body of research in different domains is lack of representativeness in study populations ([1]– [2]). Populations such as ethnic minorities ([3]– [4]), low-income populations [5], ethnic minorities in low-income countries [4, 6], participants from same-sex relationships [7], illiterate or limited literacy populations [8], immigrant, asylum seekers and refugees [9] and marginalized communities remain significantly underrepresented in the literature. These populations are considered to be living in vulnerable situations, considering the barriers they face, related to social, economic, legal, or health circumstances.

Participant diversity in research is a greater challenge within interventional studies. A review of literature shows that the rates of participation, for example, in cancer trials, are especially low for ethnic minorities [10]. The same is true for other vulnerable groups [6], AIDS clinical trials [11], Alzheimer disease research [12], and studies focusing on women’s health [13].

To reduce health disparities, it is essential to increase the diversity of research participants ([14]– [15]) and promote researchers’ openness to the importance of diversity. The critical nature of this was well underscored during the COVID-19 pandemic [2]. Although certain studies, for instance Stewart et al.‘s work [16], have sought to provide guidelines to enhance the feasibility of recruiting diverse populations, their proposed solutions predominantly focus on traditional data collection methods, that have demonstrated limited success in encompassing the broader population in research.

According to the literature, the reasons for under representativeness of people living in vulnerable situations in research studies is often attributed to researchers’ challenges in accessing these populations, difficulties in motivating them to participate, obstacles in data collection, and maintaining participant engagement throughout the study process. Ethnic minority communities are often perceived difficult to reach ([13, 17]– [18]), a perception often linked to a range of social and cultural factors [13], fear of authorities [19] and privacy and confidentiality concerns [20]. Some minority groups wish to remain unseen because of uncertainties surrounding legal or visa status [21]. In addition, refugees frequently face unstable housing conditions ([22]– [23]). They may experience homelessness, reside in temporary accommodations or forced to relocate frequently [24]. Asylum seekers often subjected to geographical isolation [25].

Researchers have used diverse strategies to reach underrepresented populations and to reduce barriers to research participation. One effective approach is to adapt methods healthcare professionals use to engage broader populations for health education and improved health practices. A good example is the use of ‘navigators,’ a strategy successfully implemented in cancer care. In this model persons from the target population are trained to disseminate knowledge and information within their communities, using their own language and communication ways. Some of the other strategies reported in the literature are cultural brokers [26–28], who bridge cultural gaps and facilitate trust and access to communities, patient navigators ([29]– [30]), who guide underserved population through healthcare systems, patient–provider language concordance or cultural and language concordant programs [31], who improves communication by their shared language and cultures, and survey guides. Although these approaches share the aim of facilitating communication, trust building, and engagement, they often stem from clinical or health system contexts and emphasize patient care or treatment navigation.

In the present study, we chose the concept of a community-based research navigator. This term was deliberately selected because it is more neutral and directly aligned with the intention of increasing participation in research rather than guiding patients through health services. Unlike terms such as ‘cultural broker’ or ‘patient navigator’, which may carry specific connotations or be tied to settings, ‘research navigator’ encompasses a range of supportive roles without implying a clinical or culturally specific function. Moreover, the population of asylum seekers and refugees is highly culturally diverse, even among people from the same country of origin. Therefore, emphasizing the cultural expertise of mediators alone would not fully capture the role they perform in facilitating research participation. The function of the research navigator is to support recruitment, facilitate communication between researchers and participants, and help overcome cultural, linguistic, and logistical barriers to participation. By using the term research navigator, we also aimed to acknowledge the community-based nature of the role and to avoid medicalized terminology that may not fully capture the purpose of engaging asylum seekers and refugees in research.

According to the UNHCR [32], refugees are people who have fled their country due to a well-founded fear of persecution based on race, religion, nationality, membership in a particular social group, or political opinion. An asylum seeker is someone who has has applied for international protection but whose claim for refugee status has not yet been determined [33].

In this study, ‘participant diversity in research’ refers to variation across social, cultural, economic, and demographic characteristics, including ethnicity, migration background, socioeconomic status, literacy, legal or visa status, and other factors that may affect participants’ access to and engagement in research.

## Aim

To explore researchers’ experiences of using community-based research navigators to facilitate participation in a research project among asylum seekers and refugees.

## Methods

### Design

This study employed a descriptive qualitative approach, as outlined by Sandelowski [34]. This method is particularly useful for exploring areas with limited knowledge and where a deeper understanding is needed. Qualitative descriptions provide straightforward accounts of phenomena in everyday terms, making them especially valuable for practitioners and policymakers [34].

The study is part of a larger project, focused on implementing and evaluating a process aimed at empowering less-researched populations such as asylum seekers and refugees. The goal was to enhance research participation among asylum seekers and refugees with Syrian, Eritrean, and Somali backgrounds by developing a community-based research navigator programme.

### Setting

The research took place in a municipality in western Sweden. This municipality is characterized by a mix of urban and rural areas and a diverse population, including relatively large population of refugees and asylum seekers at the time of the study.

We interviewed the researchers, referred to as informants in this paper, who implemented a community-based research navigators programme for the first time in a project focusing on mental health among refugees and asylum seekers. The project was a large quantitative study which aimed to assess mental health of these population, in one of the largest accommodations zones for asylum seekers in Sweden, at the time of the study. Refugees and asylum seekers in Sweden are in general placed in locations where accommodations are available across the country, at the time. Examples of such accommodations are former residential care facilities, summer cottages, old schools, and ordinary apartments.

#### The community-based research navigators’ programme

The community-based research navigators programme involved inviting volunteers from the target population of the research project consisting of Syrian, Eritrean and Somalian communities to function as community-based ‘research navigators. Volunteers were recruited by placing announcements inside the large accommodation facilities for asylum seekers, in Swedish Red Cross centres providing consultation for asylum seekers and refugees, as well as in recreational venues frequently visited by these groups. Some of the Swedish Red Cross volunteers’ group, active in the site, assisted in bridging the informant to the candidates for research navigator’s programme.

The main criteria for volunteers were the ability to read and write in Swedish or English and their native language, and to communicate well with the target population. Those who contacted the researchers to express interest in becoming community-based research navigators were subsequently provided with detailed information about the study and their potential role by telephone and letter. Thereafter, all interested volunteers were invited to a meeting with the researchers to receive further information, discuss expectations, and prepare for their role. All interested volunteers were then invited to an onsite meeting with the researchers, where they received further information about the project, expectations, and responsibilities. During this meeting, different scenarios in communicating with participants during recruitment and data collection were discussed and rehearsed. Training was provided directly by the research team and focused on ethical considerations, informed consent procedures, confidentiality, and strategies for culturally sensitive communication. One of the researchers regularly travelled to the study site to meet the navigators, discuss ongoing challenges, and provide supervision and support.

The trained research navigators distributed the healthcare project’s questionnaires, among the target populations. The research participants received the questionnaire in an envelope with the possibility to send back the questionnaire or getting it collected by the navigators. They also could have received the navigator’s assistance in reading and completing the questionnaires, if they wished. The navigators’ other responsibilities included following up completion of the questionnaires, responding to the research participants’ questions and to facilitate communication and trust between the research team and the study population. The research navigators received training to perform their responsibilities. The response rate of the research participation in target population was relatively high (47,4%), and the navigators were able to recruit nearly half of the target populations to the research.

### The informants

The informants consisted of the research team who implemented the community- based research navigators’ programme. All researchers on the research team agreed to participate in our study.

The informants included both senior and junior researchers including a doctoral student, three were men and one woman, aged between 27 and 53 years old. All except one of the researchers were of Swedish origin, one informant identified as having an Asian background. They all had experience of working with or studying people with diverse cultural backgrounds including immigrants and refugees.

The informants were stationed in one of the community services of Swedish Red Cross Organization in that municipality, for implementing the community-based research navigator programme. The location was near the camps of the asylum seekers and refugees, where some of them often spent time engaged in leisure and social activities and accessed received various forms of support including consultations. None of the informants resided in that city or surrounding area.

### Data collection

In total, eight individual in-depth interviews were conducted with the informants, who implemented the community-based research navigator programme, both before and after its implementation.

An interview guide was developed in advanced, including some open-ended questions. Examples of the questions include: From your perspective, what advantages do the use of a research navigator have compared to alternative methods such as emails or other methods? The first interviews before implementing the programme, explored the informants’ expectations and anticipated challenges before implementing the navigator programme. The second set followed a slightly adapted guide that included the same core questions but also additional probes about experiences of working with navigators after the programme had been implemented.

The first interview with each informant was carried out at their workplace which was a university setting located in one of Sweden´s major cities. The second post implementation interviews were conducted at the implementation site. The interviews lasted between 43 and 62 min, all interviews were recorded and transcribed verbatim.

Participation was voluntary and they were able to withdraw any time before the data analysis began. All informants were fully informed of the study and gave their consents to participate in written. To protect confidentiality, all participants were assigned pseudonymous names, which are used when presenting interview quotes in the results section.

### Data analysis

We conducted an inductive thematic analysis inspired by Braun and Clarke [35]. The process involved six phases: familiarising with the data, generating initial codes, searching for themes, reviewing themes, defining, and naming themes, and finally reporting.

First, the team familiarized themselves with the dataset by reading it thoroughly. Next, the first author systematically coded the data, identifying meaningful segments and assigning relevant codes. These codes were then grouped into preliminary themes based on overarching ideas. The themes were reviewed for accuracy and refined into subthemes where necessary. The research group collaboratively defined and named the themes to clarify their meaning and articulate the overall narrative. Finally, the authors drafted the manuscript to report their findings.

The analysis is aligned with the Reflexive Thematic Analysis Reporting Guidelines (RTARG) created by Braun and Clarke [36]. This was conducted with a view to enhance the quality and transparency of the analysis process, which in turn increases the methodological rigor of this study.

The authors´ describe the setting, data collection and analysis process carefully to enhance the assessment of transferability as described by Patton [37]. As suggested by Kvale and Brinkman [38] quotations were used to support our analytical findings.

## Results

The analysis resulted in two themes and five subthemes, describing the researchers’ experiences of using community-based research navigators in facilitating participation among asylum seekers and refugees in a research project on Mental Health (Table 1). The themes include ‘critical enabler of participants recruitment’ and ‘complexities of intergroup relationships and logistics.

**Table 1:** An overview of the themes and sub-themes

Critical enabler of participant recruitment *Navigators’ meaningful engagement and recognition* C*ommunity- based language concordance as a trust builder*
Complexities of intergroup relationships and logistics *Power dynamics* *Communication and relational challenges* *Logistic challenges*

### Critical enabler of participant recruitment

#### Navigators’ meaningful engagement and recognition

The informants experienced that the involvement of the navigators was of utmost importance for the navigators themselves. The informants perceived the navigators as proud and motivated to contribute to knowledge generation, with the aim of benefiting their fellow countrymen and other asylum seekers and refugees. One informant expressed:

*“It is very important that we are able to carry out this study and gain this knowledge*,* because eventually arriving at insights that may be helpful to the researcher guides fellow countrymen and to other refugees makes the researchers guides motivation essential.” (Informant Karl)*.

Participation in the study as a research navigator was voluntarily without any financial compensation. However, the navigators were offered a paid membership in the local Red Cross circle and a tote bag for storing the survey materials. At the end of the study, the navigators were awarded certificates acknowledging their involvement in the project, as a token of appreciation and recognition.

Despite the lack of financial compensation, navigators were initiative-taking, viewing their role as meaningful. One informant addressed:*” Working as a research navigator get them engaged in something that was also meaningful for them.”* (*Informant Paul).* The success of participant recruitment in the overall study depended on the motivation of both the research navigators and the target population of asylum seekers and refugees. One informant expressed: *“Although the full evaluation of the process by the researchers is still pending*,* the experience so far has been very positive.” (Informant Mikael)*.

Upon completion of their assignments, the collaboration between the informants and the navigators came to an end. When the project concluded the informants organised a meeting with the navigators to gather and discuss their experiences. During this meeting, the navigators shared valuable insights and feedback about their roles and the overall project.

The informants emphasized that navigators’ educational levels, and previous skills were crucial in enabling participation and clarifying the study’s relevance for the asylum seekers and refugees. Examples included experience of working as teacher, physician as well as having research and statistical knowledge. One informant stated:

*“Almost all research navigators were highly educated. Most of them were very well versed in how research works and what conclusions can and cannot be drawn. However*,* some research navigators belonged to the support group and did not have earlier experience of research.” (Informant Mikael)*.

According to the informants, it would have been beneficial if the navigators had some form of healthcare education and previous experience in providing psychosocial support, as part of the aim of the overall project was to inform asylum seekers and refugees about inquiring where to seek various healthcare supports. The educational backgrounds of the navigators varied significantly among the groups. Some had limited formal education, while in others had a higher university education.

The informants reported that, in their experience, it was easier to recruit asylum seekers and refugees when using navigators. The informants recruited navigators who shared the same country of origin, culture, and experiences as the asylum seekers and refugees. The navigators have either been asylum seekers themselves or are currently asylum seekers living in the same area. The informants involved this group not only to engage them in the research project but also because they participated in the survey study. The navigator’s involvement lends a certain legitimacy to the research process, helping to convey the message in a culturally appropriate manner that resonates with those who are supposed to answer the survey. Additionally, the navigators were able to help the informants to avoid misunderstandings or concerns that they might overlook due to the diverse backgrounds and experiences. The navigators could explain the terms used in describing the project, and in questionnaires, for instance what means with cohabitation, and if that is equivalent to living with girlfriend or boyfriend. They also helped validate the importance of the project.

### Community-based language concordance as a trust builder

The informants experienced integrating community-based research navigators to be a critical enabler in effectively reaching the target population of asylum seekers and refugees. The navigator’s ability to conduct home visits, information meetings and communicate in the asylum seekers and refugees’ native languages significantly increased trust and participation. The informants expressed satisfaction over their choice of assigning research navigators from the same home country and national language to assist in data collection from the respective community. The community-based research navigators, seemed to be able to raise awareness about the ongoing research project, and motivate the community to participate in the project.

The community-based research navigators were able to help the target populations to distinguish between the researchers and their intensions with the Swedish authorise such as Board of migration. This seemed to help the target group to trust the researchers in sharing information about their mental health via questionnaires. The navigators could help the target group set aside misconceptions or fears, for example, concerns that their responses in a mental health project might affect their asylum case, or that the Swedish Migration Board could be influenced by the study’s findings regarding asylum seekers’ mental health. The navigators were able to clarify the concept of confidentiality and anonymity in research and explain how the researchers planned to handle the data and ensure data integrity. The navigators have themselves had the opportunity to pose questions and discuss their wonderings with the researchers in training workshop prior to distributing the questionnaires. This helped build trust and sense of connection with the research team, which they tried to transfer to the target populations.

Experiencing an established trust and functional collaboration between the informants and the navigators, only one researcher was needed to reside on site for support the navigators.

Recruitment of asylum seekers and refugees involved significant logistical coordination, particularly for home visits. One informant expressed:

*“When we collect data at refugee reception centres*,* the research navigators must visit the people in their homes. This requires more logistical and specific resources because we feel the research navigators need to explain*,* explain much more about logistical and are about*,* so the people don’t get scared. It helps to increases the legitimacy when the research navigators can explain what they are dealing with it” (Informant Karl)*.

According to the informants, navigators’ flexibility to engage asylum seekers and refugees outside standard hours was crucial, particularly as many asylum seekers and refugees attended language classes during the day. Many asylum seekers living in the camp tended to sleep during the day (partly to pass the time) and stayed awake late in the evenings and at night. The flexibility of the navigators being available in the community and able to reach out to the target group or respond to questions was perceived as highly valuable.

Navigators were able to identify and recruit asylum seekers and refugees, guide them to relevant healthcare and support services, and participate in the research project, thanks to familiarity to their home county fellows’ culture and language. The navigators needed a comprehensive understanding of the project, Swedish society, and the healthcare system. The informant experienced a diverse level of preparedness and confidence among the research navigators, despite having recieved similar training. Some navigators were experienced to be well prepared while other navigators seemed uncertain about their roles, with a need for more tailored training. *“But then there were a few navigators who maybe …. I wondered whether we had fully reached the intended aim…. that perhaps some navigators who*,* had not fully understood the task.” (Informant Paul)*.

Before the project started, there was a hope of a large study participation. Two of the informants in this study described that there was one group of asylum seekers and refugees who were not included in the research project but wanted to participate. Asylum seekers and refugees appreciated the project for giving voice to their experiences and highlighting issues relevant to their communities.

On the other hand, some potential asylum seekers and refugees refused to join due to lack of personal benefit, disappointment with support groups at the refugee camp or recent residence permit denials. One informant stated:

*“I met an asylum seeker have just been rejected*,* the asylum seeker was too upset*,* feel so bad and really don’t want to answer this survey. Another asylum seeker said*:

*You didn’t help me with what I needed*,* but now you want help from me with this? Then I won’t help you either.” (Informant Kristina)*.

Informants stated that the navigators should be given the opportunity to provide information about participation in the study individually to potential asylum seekers and refugees. This was an important aspect as many of the asylum seekers and refugees lived with others and some of the questions in the survey can be perceived as being of a sensitive nature.

Recruiting community-based language concordance as research navigators was experienced in general quite easy. However, female navigators were harder to recruit, and in some cases, asylum seekers and refugees preferred to interact with someone of the same gender or age group. This was understood to be related to their religious beliefs which seemed not to recommend socializing with the opposite sex.

Most of the navigators were enthusiastic and inspired by their new assignments, viewing them as both important and enjoyable. One informant stated: “*To hear the research navigator’s enthusiasm about the project was very important for us.” (Informant Mikael)*.

Additionally, the informants found that the data was collected smoothly while offering the navigators something valuable to engage with. *“And then the research improves with an opportunity to reach more participants*,* while at the same time we give something back to the research navigators*,* so they feel that they are contributing to something meaningful as well.” (Informant Paul).*

### Complexities of intergroup relationships and logistics

#### Power dynamics

There were preexisting and developing power dynamics among the research navigators and in the research population. Such power dynamics were partly rooted in the communities’ cultural, political, and historical aspects and partly influenced by the age, gender and educational factors.

The informants stated that the navigators’ dual role, as both community members and representatives of the research institutions, sometimes, created tension and posed challenges to build trust in the research process. Informants concerned about the use of collected data and the safety of the asylum seekers and refugees. These dynamics were further complicated, in some cases, by the complexities of intergroup relationships, where differing expectations, power imbalances, and historical experiences with authorities or institutions influenced perceptions and interactions throughout the research engagement. In some cases, there was considerable suspicion and mistrust within different refugee groups.

Some statements indicated that differences between the navigators and the asylum seekers and refugees could complicate the work. There were situations where asylum seekers and refugees preferred to interact only with people of their own age and own sex. However, the navigators were able to decide which asylum seekers and refugees they should contact. For example, in one group, the women distributed surveys to other women. One informant stated: *“It was decided in the Syrian group that the women would distribute the questionnaires to other women.” (Informant Paul)*.

### Communication and relational challenges

Language mismatches between informants and navigators sometimes hindered communication, highlighting the need for interpreters or multilingual training. There were some navigators who did not speak English very well.

Informants also encountered ethical dilemmas in the use of navigators, which influenced their ability to foster participation in a culturally sensitive and trustworthy manner. One such dilemma involved the competition among navigators regarding the number of surveys they distributed. Another ethical concern arose from situations of friendships between asylum seekers and refugees and navigators.

According to the informants, the asylum seekers and refugee groups often came from diverse cultural and ethnic and political backgrounds and had experience various forms of displacement and trauma. Such differences contributed to complex intergroup dynamics that influenced trust and participation in the research process. There were several potential risks when using navigators from within the asylum seekers and refugees’ communities to assist with data collection. The primary concern was the issue of trust. According to the informants the asylum seekers and refugees sometimes questioned the navigator´s motives or the accuracy of the information they collected. “*There are several risks when you involve members of the group in data collection. Sometimes*,* you might not trust the navigator – it could feel too personal*,* like they´re a friend*,* or perhaps you don’t get along.” (Informant Karl)*.

### Logistic challenges

According to the informants, the navigators had difficulties locating participants due to inaccurate address lists provided by the Board of Migration. Some people listed at certain addresses could not be located, and some residents were not registered on the official Board of Migration ‘s address records. In addition, some asylum seekers and refugees had relocated from the reception centres and others had been sent back to either the first country of arrival in the EU or to their home countries.

According to the informants, some asylum seekers and refugees may have provided socially desirable responses, possibly influenced by fears related to their asylum status. For example, there was a feeling that the asylum seekers and refugees gave false answers to increase their chances of being allowed to stay in Sweden. Furthermore, there was a fear among one of the groups that they were not loyal to the regime in their home country and that their responses to the survey could be reported to the regime in their home country.

Informants expressed differing views on whether the time allocated for recruiting asylum seekers and refugees was sufficient to ensure participation. Some informants felt that the time allocated for data collection was too limited to achieve the desired recruitment goals. Additionally, cultural, and religious events played a significant role in hindering the navigators to inform and recruit asylum seekers and refugees. One informant expressed that scheduling conflicts with religious practices hindered recruiting asylum seekers and refugees for participating in the overall project: *“we happened to book in an information meeting during the prayer time*,* it was their prayer time” (Informant Mikael).*

## Discussion

The present study explored researchers’ experiences of community-based research navigators’ involvement in facilitating research participation. The findings underscoring the valuable contribution of community-based research navigators as critical enable of participants recruitment and research participation, while also highlighted experiences of a complex landscape shaped by complexities of intergroup relationships and logistics, including the preexisting and developing power dynamics, as well as communication and relational challenges.

The constructive contribution of community-based research navigators was highlighted. The process of recruitment was described as smooth and meaningful with the research navigators demonstrating a high level of independence where the navigators took own responsibilities for recruiting participants. This is in keeping with Hearn et al. [39] who highlight how community researchers can help build trust to research within communities while also enhancing researcher´s understanding of the communities. Working with community-based research navigators have the potential to be applied in other contexts and other groups. Training community members to participate in data collection enhances the accessibility and cultural sensitivity of the research project. It is also regarded as a means of creating trust and rapport with asylum seekers and refugees but also contributes to more accurate and contextually based data. The findings highlight the importance of an awareness of intergroup relations among refugee communities. Intergroup relations were impacted by cultural background, political beliefs, etc. They ranged from expressions of community solidarity based on shared experiences of displacement, to expressions of distrust and suspicion which could led to reluctance to engage due to concerns about surveillance or disclosure of sensitive information.

Contextual constraints were shaped by the often-unpredictable nature of the research field, the availability of asylum seekers and refugees, and the potential emotional toll of conducting assessments in high stress environments. This included participants showing varying levels of preparedness to engage with the study alongside shifting demographics among groups. These challenges were partly due to inaccurate address lists provided by the Board of Migration and a degree of mistrust and suspicion toward the project. These forementioned constraints/factors required a sensitive and responsive approach to asylum seekers and refugees’ recruitment and data collection. Robinson et al. [40] highlight the importance of acknowledging, in health care, ‘the many stress factors’ which shape the experiences of persons in forced migrant communities. Establishing trust requires the presence of safe, welcoming spaces and can often only begin to develop after multiple encounters. A researcher can build trust by communicating openly about goals and challenges, showing appreciation for the community-based research navigator’s expertise, and engaging actively in the collaboration. Following up on advice, being transparent about progress, and involving the navigator early in the process also help. Trust grows over time through consistent interaction, mutual respect, and shared commitment to the research’s success.

Other contextual considerations emerged from the informants` experiences as the project evolved, included a recognition of the need to expand the survey that the asylum seekers and refugees by participating in the large quantitative study on mental health among asylum seekers and refugees in Sweden. Having an insider perspective with a more comprehensive understanding of the asylum seekers situation provided opportunities to identify other areas of interest and urgency. However interesting, this in turn could conflict with the limitations of the current project. This study underscored the importance of acknowledging the heterogeneity and the complex intergroup dynamics within refugee communities and. the need for more individualised approaches to asylum seekers and refugees’ engagement. Individual approaches could be for example tailored education in research methods for community-based research navigators. This principle extends beyond research and applies broadly across different contexts in society. Spaaij et al. [41], in their work on refugee participation in sport, emphasise the value of intersectionality in acknowledging the complexity of identity. Their findings reinforce the need to tailor engagement strategies that are responsive to the diverse needs and lived experiences of refugees. In this study, asylum seekers and refugees are categorised after the land in which they come from.

The gender and age differences between the community-based research navigators and the asylum seekers and refugees may highlight the need to adjust assigning navigators to the research population, to ensure more sensitive and effective engagement. In Bekteshi et al. [1] research study, recruiting of Afghan women proved challenging due to cultural norms that often require them to be accompanied by male family members. This in turn could inhibit the women´s willingness to join the study and impact upon their willingness to respond freely. It was suggested how this outcome could be addressed by employing more female research navigators.

The findings of this study offer insights into best practices and areas for future inquiry in the field. The involvement of research navigators in refugee mental health research may raise several ethical and relational complexities. While navigators help bridge cultural and language gaps, they may find themselves in ambivalent positions, embedded within both the community and the research infrastructure. This potential duality can generate power imbalances and positionality issues. Forced migrants acting as navigators, though essential in recruitment, may still be situated in vulnerable positions, raising the risk of exploitation or tokenism. Ethical guidelines caution against overprotecting vulnerable contributors while simultaneously compromising their autonomy and agency [42]. Table 2 provide suggestions for best practices for engaging community-based research navigators in refugee mental health studies. These recommendations underscore the importance of culturally congruent recruitment, structured and context-sensitive training, adherence to ethical standards, and sustained collaborative partnerships to ensure methodological rigor and participant trust.

**Table 2:** Best practices working with community-based research navigators in refugee mental health research

• Recruit from within the community - Select navigators who share cultural and linguistic backgrounds with participants to build trust and improve communication.
• Provide tailored training - Equip navigators with clear guidance on research ethics, roles, and procedures, adapted to their educational and experiential levels.
• Respect cultural and gender norms - Match navigators and participants by gender and age when appropriate to ensure culturally sensitive engagement.
• Maintain ethical boundaries - Address potential conflicts from personal relationships and power dynamics to protect data integrity and participant autonomy.
• Foster ongoing collaboration and recognition - Engage navigators early, value their input, and acknowledge their contributions to sustain motivation and trust.

Ethical dilemmas may arise in projects of this kind. Personal relationships could compromise the integrity of the survey process, as it might be challenging for the navigators to deny participation to their friends. On the other hand, if asylum seekers and refugees had a negative relationship with the navigator, it could lead to biases and conflicts that hinder effective data collection. Additionally, there was a significant risk that navigators could inadvertently assume positions of power over asylum seekers and refugees. Confidentiality also becomes complex when navigators know participants personally or share social networks. Maintaining privacy and integrity, may become psychologically and socially burdensome for navigators, requiring robust ethical oversight. Additionally, dual relationships, where navigators serve peers who are also friends, may present ethical tensions around consent, undue influence, and boundary management. Such nuances are highlighted in ethical literature that stresses the importance of relational ethics and negotiating ethical conduct beyond procedural norms ([43]– [44]). Trust is another central component in relationships among research navigators, research participants and researchers. Refugee research ethics emphasize respectful engagement and avoiding intellectual harm that is, not framing participants as mere objects of study but acknowledging their knowledge and capacities. Navigators may cultivate trust but also may unintentionally reinforce power asymmetries if not fully supported or positioned transparently.

The findings of this study highlight the critical importance of navigator’s language skills when participating in a community-based research navigator programme. Language skills can enable clearer communication and establish trust which in turn can enhance asylum seekers and refugees’ involvement. Our findings are compatible with the framework of patient provider language concordance as described by Hsueh et al. [31].

Furthermore, navigators may experience emotional burden, as they relay difficult information or manage expectations in settings where participants may face precarious legal or social circumstances. Ensuring support structures for them and mental health resources aligns with ethical frameworks that call for caring for those facilitating research, not only those being researched [45].

Lastly, ethics guidelines for research on asylum seekers and refugees, call for tailored protections that go beyond generic standards, attending to layered vulnerabilities in forced migrant populations stemming from trauma, uncertainty, and unstable environments. When navigators interact with individuals in these settings, the ethical responsibility of researchers extends to creating safe, respectful, and sustaining engagement structures.

The community-based research navigator programme showed to be a valuable yet complex strategy for facilitating research participation among asylum seekers and refugees. This approach holds promise for improving inclusivity and equity in health research involving hard-to-reach populations, such as asylum-seekers and refugees. Involving asylum seekers and refugees in research helps ensure that research outcomes more accurately reflect the health needs, priorities, and lived experiences of these populations, which are often underrepresented in health studies. Such involvement may also enhance trust in research within these communities, reduce barriers to participation in future studies, and contribute to the development of more equitable and culturally sensitive health interventions and policies. In the longer term, this approach has the potential to strengthen health systems by generating knowledge that is relevant for both migrant and host populations.

### Limitations

Several aspects need to be considered when interpreting the findings. The insights gained are context specific and may not reflect broader experiences across different settings or populations. While qualitative research aims to provide depth rather than breadth, the limited number of informants may have constrained the diversity of perspectives captured.

The dual role of the informants, as both project designers and informants, introduces potential bias. Their involvement in planning, implementing, and evaluating the research navigator programme may have influenced their reflections during the interviews. However, the relatively high participation rate in the mental health research project (47,4%) following implementation of the community-based research navigators programme provides an objective measure, indicating that research participation was indeed facilitated.

The study relied on subjective accounts of participants, which may be subject to recall bias. Informants’ perceptions of the programme’ s effectiveness and challenges might have been shaped by the outcomes of the project or by their own expectations and assumptions.

Another important aspect, not raised by the informants in this study but worth considering, is that the target populations in such research are socially and structurally vulnerable. Selecting some community members as navigators may grant them social capital and influence that could potentially be used for purposes other than the research. This is a delicate decision that researchers need to make carefully, alongside close monitoring of the programme’s implementation and remaining available to the target group to address any emerging issues or potential misuse of these power relationships.

## Conclusion

This study highlights the potential and complexity of employing community-based research navigators to facilitate participation among asylum seekers and refugees in a health research project. The findings underscore that navigators, people with shared cultural and experiential backgrounds, can serve as enablers in bridging gaps between researchers and target populations. The research navigators’ involvement may assist in fostering trust, improving communication, and facilitating access to participants who are often underrepresented in research. However, the study also reveals the nuanced challenges that accompany such strategy. These include logistical constraints, intergroup dynamics, communication and ethical challenges. The dual role of navigators as both community members and research facilitators may introduce complexities that required careful navigation to maintain trust and research integrity. To maximise the effectiveness of such programmes, future initiatives should consider well-tailored training for navigators, sensitive recruitment strategies, monitoring the implementation and mechanisms to address ethical concerns. Moreover, acknowledging the vast heterogeneity within asylum-seekers and refugee populations and adopting individualised, culturally responsive engagement strategies are essential for fostering inclusive and ethical research practices. The community-based research navigator programme offers a promising, community empowered approach to improving research inclusivity. When thoughtfully implemented, it not only enhances data quality and participation rates but also contributes to more equitable and representative research outcomes.

## Data Availability

The data that support the findings of this study are available on reasonable request from the corresponding author. The data are not publicly available due to ethical restrictions as they contain information that could compromise the privacy of informants.
