# Correction: Facilitating research participation through a research navigator programme: researchers’ insights from a project with asylum seekers and refugees

**DOI:** 10.1186/s13031-025-00747-0

**Published:** 2026-01-27

**Authors:** Hägg Martinell A., Tyrrell M., Eriksson H., Mazaheri M.

**Affiliations:** 1 Department of Health Promoting Science, Sophiahemmet University, Stockholm, Sweden; 2 Department of Nursing Science, Sophiahemmet University, Stockholm, Sweden; 3 Department of Neurobiology Care Sciences and Society, Karolinska Institutet, Stockholm, Sweden; 4 Department of Health Sciences, University West, Trollhättan, Sweden

**Correction: Conflict and Health (2025) 19:88**

10.1186/s13031-025-00729-2.

In this article the citation was incorrectly given as “A., H.M., M., T., H., E. et al. Facilitating research participation through a research navigator programme: researchers’ insights from a project with asylum seekers and refugees. Confl Health 19, 88 (2025). 10.1186/s13031-025-00729-2.” but should have been “ Hägg Martinell, A., Tyrrell, M., Eriksson, H. et al. Facilitating research participation through a research navigator programme: researchers’ insights from a project with asylum seekers and refugees. Confl Health 19, 88 (2025). 10.1186/s13031-025-00729-2.”.

The original article has been corrected.

